# Spontaneous focal activation of invariant natural killer T (iNKT) cells in mouse liver and kidney

**DOI:** 10.1186/1741-7007-8-142

**Published:** 2010-11-30

**Authors:** Jia Zeng, Jonathan C Howard

**Affiliations:** 1 Institute for Genetics, University of Cologne, Zuelpicher Strasse 47a, 50674 Cologne, Germany; 2 Section of Experimental Therapeutics, Leeds Institute of Molecular Medicine, Level 7, Wellcome Trust Brenner Building, St James's University Hospital, Leeds LS9 7TF, UK

## Abstract

**Background:**

Invariant natural killer T (iNKT) cells differ from other T cells by their hyperactive effector T-cell status, in addition to the expression of NK lineage receptors and semi-invariant T-cell receptors. It is generally agreed that the immune phenotype of iNKT cells is maintained by repeated activation in peripheral tissues although no explicit evidence for such iNKT cell activity *in vivo *has so far been reported.

**Results:**

We used an interferon (IFN)-γ-inducible cytoplasmic protein, Irga6, as a histological marker for local IFN-γ production. Irga6 was intensely expressed in small foci of liver parenchymal cells and kidney tubular epithelium. Focal Irga6 expression was unaffected by germ-free status or loss of TLR signalling and was totally dependent on IFN-γ secreted by T cells in the centres of expression foci. These were shown to be iNKT cells by diagnostic T cell receptor usage and their activity was lost in both CD1 d and Jα-deficient mice.

**Conclusions:**

This is the first report that supplies direct evidence for explicit activation events of NKT cells *in vivo *and raises issues about the triggering mechanism and consequences for immune functions in liver and kidney.

## Background

Invariant natural killer-like T (iNKT) cells are placed ambiguously between adaptive and innate immune systems (reviewed in [1,2]). Derived from the thymus, expressing rearranged T-cell receptor (TCR) alpha and beta chains, they seem to belong to the adaptive immune system, while their receptor homogeneity, their continuous state of activation, their rapid secretion of large amounts of interferon (IFN)-γ and interleukin (IL)-4, their presumed recognition of invariant glycolipid self-ligands associated with the non-classical major histocompatibility (MHC) class I molecule, CD1 d, recall various aspects of innate immune recognition. Many features of iNKT cell behaviour are puzzling: their thymic ontogeny and relation to the classical pathways of T-cell mediated differentiation; the relative importance of endogenous and exogenous ligands in activation; and the polarity of their cytokine profile towards IFN-γ or IL-4 in relation to the activating ligand. However, in this report we address the basis for another characteristic of these enigmatic cells, namely their constitutive state of readiness to respond with massive cytokine production (reviewed in [3]). Using a sensitive endogenous reporter for IFN-γ production, we show that iNKT cells are constitutively and endogenously activated to IFN-γ production in the liver and kidney of normal mice. The activation is apparently restricted to these tissues, focal, spontaneous and independent of signals derived from bacteria or viruses. It is, however, dependent on the expression of CD1 d and on the presence of the classical iNKT cell receptor. The results suggest that the constitutive state of iNKT readiness may be maintained by intermittent local stimulation with endogenous ligands.

## Results and discussion

**Figure 1:**
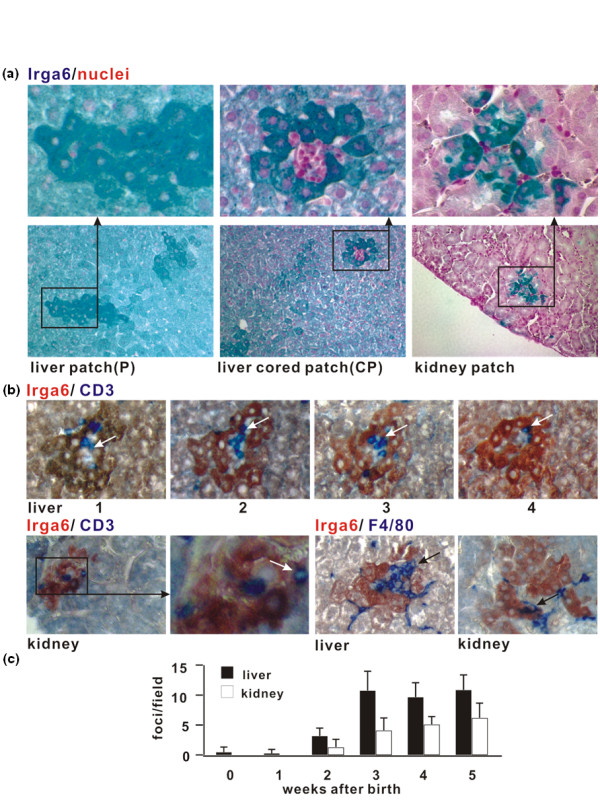
**Focal expression of Irga6 in healthy mouse liver and kidney**. (A) Irga6 is expressed focally in the liver and kidney. Paraffin sections of C57BL/6 healthy adult mice liver and kidney were probed for Irga6 protein (green). In liver the parenchyma, between the high expression foci, is stained uniformly with Irga6 as a result of transcription from the liver-specific Irga6 promoter [4]. Two types of Irga6 focal expression in liver are depicted: a stained patch without an evident core (P) and a patch with a mononuclear cell core (cored patch, CP). Here and in (B) the microscope magnification is 200×; frames show enlargements. (B) T cells and macrophages/dendritic cells are present in the mononuclear cores of the liver and kidney patches. Wax sections of the livers and kidneys from adult C57BL/6 mice were stained for Irga6 (brown/red) and CD3 or F4/80 (blue). For Irga6 and CD3 double staining in liver, consecutive serial sections were analysed (numbered 1, 2, 3, 4). Arrows point to CD3 and F4/80 positive cells; frames show enlargements. (C) Irga6 focal expression is developmentally regulated. Wax sections through whole livers and kidneys from C57BL/6 newborn mice and mice, aged as indicated, were stained for Irga6. High expression foci were counted within multiple microscopic fields (see Methods section). Each value in the histogram represents the mean of 30 such counts for organs from two mice of each age (error bars indicate standard deviations).

We recently demonstrated the constitutive expression of the IFN-γ-inducible, immunity-related GTPase, Irga6, in hepatic parenchymal cells of normal mice [4]. This was attributed to the presence of a dedicated, liver-specific promoter associated with this innate immune resistance gene. During these studies we noticed that the expression of Irga6 in hepatic parenchymal cells was not uniform. Small focal patches, each consisting of a few to a few dozen contiguous cells, expressed very much more Irga6 than the general expression level (Figure 1A). These foci of high Irga6 expression resembled those that we reported in the kidney parenchyma associated with tubular epithelium [4]. About 10% of high expression foci in the liver were characterized by a central accumulation of small mononuclear cells. We could establish immunohistologically that T cells, defined by CD3, and macrophage/DC lineage cells, defined by F4/80, were present in these mononuclear cell cores (Figure 1B). The same cell types could be found adjacent to the patches of high Irga6 expression in the kidney cortical tubular epithelium, following closely the pattern of F4/80+ DC described in mouse kidney [5]. In subsequent analysis of the liver we distinguished between the minority of Irga6 expression patches with and the majority without, visible mononuclear cell cores (see Materials and Methods), as they clearly have different origins (see below). There are mononuclear cells associated with all kidney patches and the patches seem to have only one origin (see below). High Irga6 expression patches are not present in newborn mice but develop rapidly in both the kidney and liver between 1 week and 3 weeks after birth (Figure 1C).

**Figure 2:**
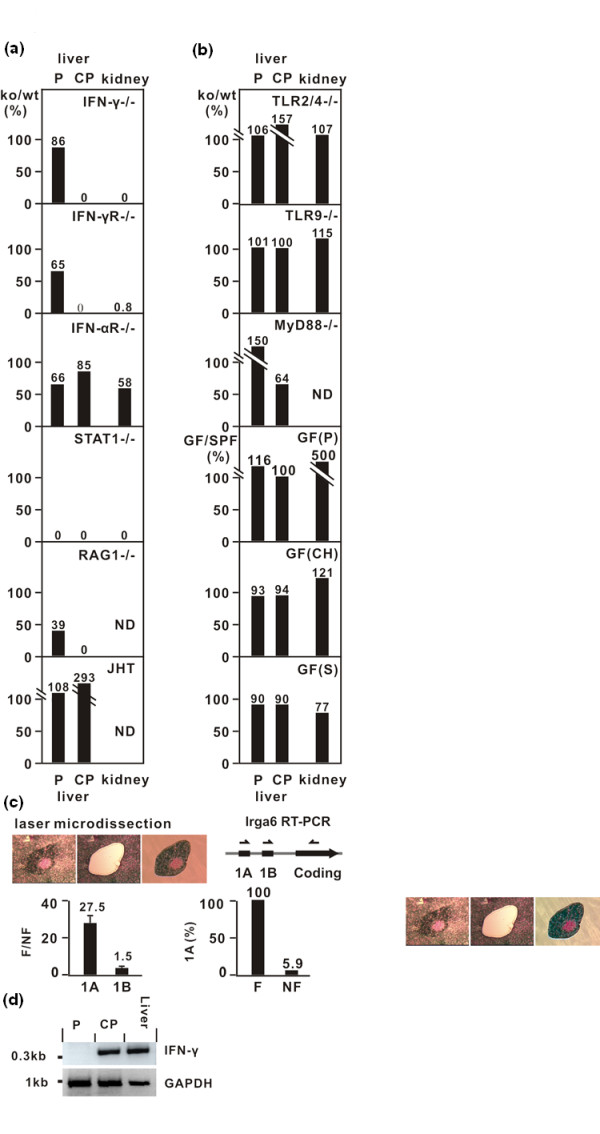
**Focal expression of Irga6 is dependent on interferon (IFN)-pathway**. (A) Focal expression of Irga6 is eliminated in mice deficient in the IFNγ-response pathway. High Irga6 expression foci were counted in livers and kidneys from two mice deficient in IFN-pathway components and from two appropriate control wild-type (WT) mice as described (see Methods section). Focus numbers from knockout (KO) organs are presented as a percentage of the focus numbers in control WT mice (ko/wt). For raw data see Additional file 1: Table S1. Kidneys of RAG^-/- ^and JHT strain could not be analysed (ND), because of generally elevated Irga6 expression (Additional file 2: Figure S1). (B) Irga6 focal expression is not triggered by immune responses to microbial components. Irga6 focal expression was assayed in organs from mice deficient in TLR2/4 and TLR9, MyD88 and germ-free (GF) mice. GF mice came from three different sources [GF(P) Portugal, GF(CH) Switzerland, GF(S) Sweden]. (C) IFNγ-inducible transcripts of Irga6 (1A) dominate focal expression in liver. IFNγ-inducible (Irga6-1A) and liver-specific (Irga6-1B) transcripts were quantitated by quantitative real-time polymerase chain reaction (RT-PCR) from focal (F) and non-focal (NF) material isolated from stained tissue sections by laser microdissection (top left: left panel, liver section before microdissection of cored patch; middle panel, after microdissection; right panel, microdissected patch). Irga6-1A and Irga6-1B transcripts were amplified using selective primers for the 1A and 1B 5'-exons and a common 3' primer in the coding exon (top right). The ratio of expression of 1A to 1B is given for F and for NF (bottom left) as the mean of two independent experiments (error bars indicate standard deviations). Twenty clones amplified for Irga6-1A from both F and NF were sequenced. The dominance of Irga6-1A in F is very high since only 5.9% of clones amplified for 1A from NF liver were specific for Irga6-1A, compared with 100% from F (see Methods section). (D) IFN-γ is expressed in liver cored patches. Total liver tissues (liver), Irga6 liver patches (P) and CP were collected as described (C). RT-PCR was carried out using IFN-γ specific primers and the products run in an agarose gel. GAPDH was used as control.

As Irga6 expression is induced by IFN-γ [6,7], we asked whether high expression patches were due to the local expression of IFN-γ. First, we examined the liver and kidneys of mice with genomic disruptions of components of the IFN response mechanism on the C57BL/6 background (Figure 2AAdditional file 1: Table S1. Both IFN-γ [8] and IFN-gamma receptor (IFNGR) [9] deficiencies eliminated all high Irga6 expression patches from the kidney and all Irga6 patches with mononuclear cores from the liver. The liver patches without cores were not significantly affected. IFNAR deficiency [10] had no marked effect on either organ. As expected, all kidney patches and liver patches with mononuclear cores were eliminated by STAT-1 deficiency. Surprisingly, the high expression patches without mononuclear cell cores were also absent in the STAT-1-deficient livers, even though the loss of type I and type II IFN receptors had no effect on them. We conclude that coreless liver patches are caused by the local action of another cytokine probably not of lymphoid origin, perhaps IFN-λ (IL-29) or IL-27 which both transduce signals via STAT1 in hepatocytes, but initiate signalling through distinct receptors [11,12]. Rag1 deficiency [13] behaved like IFN-γ or IFNGR deficiency, eliminating all Irga6 high expression foci with mononuclear cores from the liver, showing that lymphocytes with rearranged receptor chains were required for the focal expression patches with mononuclear cell cores but not for the coreless patches. It was not possible to assess high expression patches in kidney because of near universal high expression in these organs (Additional file 2: Figure S1). No liver phenotype was detected in B-cell-deficient JHT mice [14], excluding B cells from further consideration. Again, excessive high expression was found in the kidneys (Additional file 2: Figure S1).

Secondly, if IFN-γ is responsible for the focal induction of Irga6, there should be activation in the high expression patches of the IFN-γ-dependent promoter of the Irga6 gene, with the use of exon 1A, in contrast to the general hepatic parenchyma in which the liver-specific promoter is constitutively expressed with usage of exon 1B [4]. We therefore isolated high expression patches and areas of general expression level by laser microdissection and used quantitative real-time polymerase chain reaction (qRT-PCR) in order to identify the use of the IFN-γ-dependent 5'-untranslated 1A exon and the constitutively expressed 5'-untranslated 1B exon in the two tissue sites (Figure 2C). Expression of the IFN-γ-dependent exon 1A was 27.5× higher by qRT-PCR in the high expression patches than in the general liver, while 1B exon expression was not altered (1.5×). However, when the Irga6-1A PCR products from the general liver were cloned and sequenced, only 5.9% of were specific (a consequence of the very low absolute expression of the IFN-γ-inducible transcript in non-focal hepatic parenchyma), while 100% of those cloned from the high expression patches were specific. Thus, the true excess of Irga6-1A in the high expression patches relative to the general parenchyma was 100 × 27.9/5.9 or about 470×, confirming the intense local induction by IFN-γ.

Thirdly, if IFN-γ is being secreted locally, affected cells should express not only Irga6 but also other members of the IFN-γ-inducible IRG protein family that are not constitutively expressed in hepatic parenchymal cells [4]. We therefore stained adjacent histological sections of liver and kidney for Irga6 and for Irgm3. Indeed, focal expression of Irgm3 coincided accurately with the high Irga6 expression patches (Additional file 3: Figure S2).

Direct evidence for the expression of IFN-γ in the patches with mononuclear cell cores, but not in the coreless patches, was obtained by RT-PCR for IFN-γ transcripts in microdissected patch material. A strong signal for IFN-γ was recovered from only the patches with mononuclear cell cores (Figure 2D). These experiments established beyond doubt that the liver and kidney contain reactive foci of local, T-cell-dependent IFN-γ production identified by the local high expression of Irga6.

The character of the small IFN-γ-secreting reactive foci in the liver and kidney suggested that they could be due to local immune activity stimulated by microbial material. We therefore analysed the liver and kidney for reactive foci from mice deficient in components of the Toll-le receptor (TLR) system (Figure 2B). No effects were seen in mice deficient in TLR2, 4 or 9 or MyD88, arguing against bacterial components as the initiators of the foci. In order to test this conclusion further, we examined the liver and kidney from germ-free mice from three independent sources. No significant reduction in numbers of reactive foci in liver or spleen was detected (Figure 2B). Bacterial infection is, thus, unlikely to be a stimulus for the reactive foci

**Figure 3:**
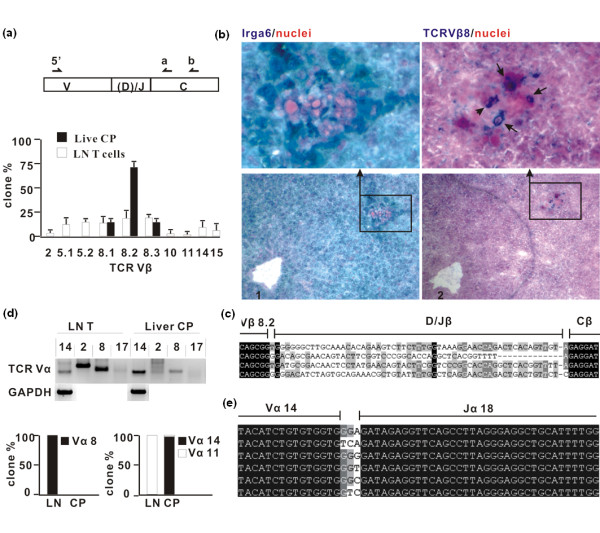
**Semi-invariant T-cell receptor (TCR) Vβ8:Vα14-Jα18 is preferentially used by T cells in Irga6 liver cored patches**. (A) TCR Vβ8 is used preferentially in Irga6 liver cored patches. TCR Vβ sequences were amplified by nested-real-time polymerase chain reaction (RT PCR) and sequenced from microdissected cored patches as described (Methods). Lymph node cells (LN) served as control. In total 68 and 132 Vβ clones for liver cored patch (CP) and lymph node (LN) T cells were identified, respectively. The histogram shows the distribution of sequenced clones among Vβ subfamilies as % of total (error bars indicate standard deviations). (B) T cells in Irga6 liver cored patches express TCR Vβ8. Consecutive cryo-sections of C57BL/6 adult mice liver were probed for Irga6 protein (1, green) and for TCR Vβ8 protein (2, blue), respectively; frames show enlarged images. Arrows indicate T cells expressing Vβ8 TCR. Microscope magnification is 200x. (C) TCR Vβ8 chains in liver cored patches have variable junctional regions, shown in alignment of TCR Vβ8.2 junctions from (A) above. Junction sequences for all Vβ8 clones from liver cored patches are given in Additional file 5: Table S3. (D) TCR Vα14 dominates Vα usage in Irga6 liver cored patches. TCR Vα usage was assessed by amplifying Vα14, 2, 8 and 17 individually from cDNA of liver cored patches or lymph node with nested-RT PCR (see Methods section: upper panel). GAPDH served as control. RT-PCR products of Vα8 and Vα14 were cloned and sequenced. One hundred per cent of Vα8 PCR products from LN T cell were Vα8, whereas 100% of the Vα8 products from liver CP were non-specific. For liver CP, 70 out of 72 clones from the Va14 PCR were Vα14; two clones were Vα11. For LN all 40 identified Vα14 clones were Vα11. The histograms (lower panel) show the corrected distribution of Vα8 and Vα14/11 usage. (E) Alignment of representative TCR Vα14/Ja18 junction regions found in liver cored patches. For full junction information on all Vα14 and Vα11 clones see Additional file 5: Table S3.

Two further explanations were considered for the focal IFN-γ production. One was the local re-expression of an endogenous mouse mammary tumour virus (MMTV) open reading frame acting as a superantigen (reviewed in [15]). The other was local activation of iNKT cells. These two possibilities could be distinguished by a knowledge of the V gene usage of T cells in the mononuclear cores. In the case of a superantigen, specific Vβ families should be favoured, but no specific Vα usage, while in iNKT cells, a specific and diagnostic pattern of Vβ8/Vα14Jα18 could be anticipated [16]. We used laser microdissection to isolate and pool the mononuclear cell cores from many high Irga6 expression patches and employed semi-nested PCR on reverse-transcribed cDNA to amplify, and subsequently clone, Vα and Vβ from expressed TCRs. The result for Vβ usage was striking, namely, the exclusive usage of the three Vβ8 genes, with an excess of Vβ8.2 (Figure 3A and 3C) in contrast to the diversity of Vβ genes recovered by the same technique from lymph node lymphocytes. We were able to confirm the high use of Vβ8 in mononuclear core T cells immunohistologically with a specific anti-Vβ8 monoclonal antibody (Figure 3B). Vβ8 is uncommon, but not unknown, as a MMTV superantigen target [15] but is part of the diagnostic receptor combination of iNKT cells [16]. We therefore amplified by RT-PCR a number of specific Vα families from lymph node cells and from mononuclear cell cores (Figure 3D). In lymph node T cells a weak signal for Vα14 was seen, compared with strong signals for Vα2 and Vα8. From the mononuclear core cells a strong signal was seen for Vα14, an unclear result for Vα2 and a weaker signal for Vα8. However, the true excess of Vα14 in the core cells was striking since the weak signal of Vα14 from lymph nodes proved, on cloning and sequencing, to be due, with a single exception, to the amplification of Vα11 which is closely related to Vα14. In contrast, all except one of the clones amplified for Vα14 from mononuclear cell cores of liver patches were indeed Vα14 (Figure 3D). Thus, the TCR V-gene usage of the mononuclear cell cores was consistent with the TCRs of iNKT cells. This was further confirmed by showing that exclusively the canonical Jα18 was used with no length variation (Figure 3E).

**Figure 4:**
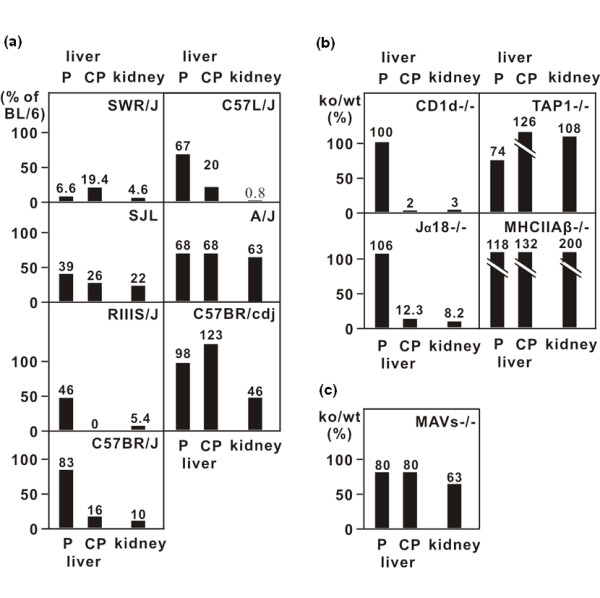
**Irga6 focal expression is reduced in mouse strains with genetic defects affecting invariant natural killer T cells (iNKT) differentiation**. (A) Irga6 focal expression was assayed in organs from mouse strains carrying natural deletions in the T-cell receptor (TCR) Vβ8 locus - namely, SWR/J, SJL/J, C57BR/J, C57L/J and RIIIS/J, A/J and C57BR/cdJ strains - that have an intact TCR Vβ locus, were also tested. Quantification of Irga6 expression foci was carried out as described (Figure 2A). Focus numbers from the test strains are presented as a percentage of the numbers found in C57BL/6. (B and C) Irga6 focal expression was assayed for organs from mice deficient in CD1 d, Jα18, TAP1, MHC II Aβ or MAVs. Quantification for Irga6 focal expression was carried out and presented as described in Figure 2A. For raw data see Additional file 1: Table S1.

If the NKT function is dependent on a dedicated Vβ8/Vα14 TCR and is responsible for the high expression patches in liver and kidney, these should be reduced in mice carrying deletions in the TCR Vβ gene array that include the Vβ8 family of sequences [17,18]. We analysed the numbers of liver core patches and kidney high expression patches in inbred mouse strains of known TCR Vβ genotype, in each case comparing patch frequencies with C57BL/6. Tissues from the A/J and C57BRcdJ strains, with normal Vβ8 gene representation, were similar to C57BL/6 (Figure 4A). However, five strains with Vβ8 deletions (SWR/J, SJL/J, RIIIS/J, C57BR/J and C57L/J) all showed reduction down to a more or less complete loss of cored liver patches and kidney patches. The residual kidney and cored liver patches in these strains may, perhaps, be attributed to usage of Vβ2 and Vβ7, V-genes that also contribute to a minority of iNKT receptors [19], although we did not find these subgroups in our analysis in C57BL/6 (Figure 3A). SWR mice had an unexplained deficit of plain liver patches.

If the Irga6 high expression foci with mononuclear cores associated with high IFN-γ production were, indeed, due to iNKT cells, they should also be absent from mice deficient in the essential ligand-presenting molecule CD1 d [20,21] and in mice deficient in iNKT cells as a result of deletion of the diagnostic Jα18 T cell receptor segment [22]. Both these predictions were fulfilled, with a substantial loss of high Irga6 expression patches from the kidney and cored patches from the liver in both mutants (Figure 4B). The patches of high Irga6 expression in the liver without mononuclear cores were not affected in either case. In order to find out whether CD4^+ ^or CD8^+ ^T cells play any additional role in the induction of high Irga6 expression patches, organs from mice lacking MHC Aβ^b ^(CD4^+ ^T cell deficient [23]) and TAP1 (CD8^+ ^T cell deficient [24]) were also examined. Patches of high Irga6 expression were normal in liver and kidney from both strains. Thus, iNKT cells alone appear to be responsible for the high Irga6 expression foci.

We present direct evidence for the constitutive activation of mouse iNKT cells in two organs, the liver and the kidneys, generating small foci of high IFN-γ expression detected by the local high expression of an IFN-γ-inducible cytoplasmic protein, Irga6. In a previous study, we surveyed the pattern of constitutive Irga6 expression in a number of other organs and high expression foci were not seen [4]. The liver and kidneys thus appear to be special in this respect. Our results suggest a local activation event that triggers production of type II interferon by iNKT cells. The mouse liver sinusoids are known to contain a major population of NKT cells and NKT cells have been reported in the kidney, probably corresponding to the T cells identified in the tubulo-interstitial spaces [25]. Dendritic cells (DC) and hepatic stellate cells (Ito cells) [26] can both present exogenous α-GalCer via CD1 d to iNKT cells. Stellate cells of the liver sinusoids have also been shown to be IL-15-dependent activators of iNKT cells to proliferation in the absence of exogenous antigen [26]. An apparently similar cell type is also found in the tubular cortex of the kidney essentially co-localizing with dendritic cells [5,27]. Both DC and stellate cells are strong candidates for the activating cell implied by our experiments, but this does not, by itself, account for the focal triggering event. Our experiments with germ-free and TLR-deficient mice make bacterial products acting on DC TLRs [28,29] an unlikely cause. More plausible would, perhaps, be a locally reactivating endogenous retrovirus triggering DC or stellate cells and, thereby, iNKT cells locally. Arguing against a primary viral stimulus, we could show no deficit in liver cored high expression patches or kidney patches in organs from mice deficient in MAVS/CARDIF [30], the common activation pathway of the cytosolic RIG helicases that act as viral sensors (Figure 4C).

It will be interesting to know how the spontaneous activation of iNKT cells and consequent constitutive IFN-γ production in liver and kidney of major inbred strains of mouse affects immunological function in these organs. NKT cells have been identified very recently as a minority population among normal resident lymphocytes in mouse kidney and both an excitatory role in ischaemia-reperfusion injury [31] and an inhibitory role in experimental glomerular nephritis induction by anti glomerular basement membrane antibody [32,33] have been described. Likewise, the large population of NKT cells that patrol the liver sinusoids [34] have been implicated in various liver disorders [35], but their spontaneous, focal activation has not been reported. Spontaneous focal activation of NKT cells in the liver and kidney with high local levels of IFN-γ will have to be considered in future studies of immune function in these organs.

## Conclusions

Invariant natural killer T (iNKT) cells are activated spontaneously in mouse liver and kidney. These activation events are local, probably extravascular, and result in the local secretion of IFN-γ. This in turn results in the local induction of IFN-γ-responsive genes in non-immune tissue cells, namely hepatic parenchymal cells and renal tubular epithelial cells. These activation events are spontaneous in the sense that they appear to be independent of exogenous pathogenic material of bacterial or viral origin. The cause of individual activation events is still unclear. In general, however, the results are consistent with a widely held view that iNKT cells are rendered spontaneously active by interaction with an endogenous ligand. In this study we demonstrate these activation events histologically. Further investigations may reveal the nature of the stimulatory event that lies at the heart of each activating focus. It will be important to generalise these findings to the human case, and to assess the implications of these findings for liver and kidney immunopathology.

## Methods

### Mice and organ preparation

C57BL/6J mice were obtained from the specific pathogen-free mouse facility in the Institute for Genetics Uni. Köln. We are enormously indebted to the many colleagues and institutions that provided mice or organs for this study. TLR2^-/- ^[36], TLR4^-/- ^[37], TLR9^-/- ^[38], TLR2/4^-/-^[39] and MyD88^-/- ^[40] mice were provided by Professor Dr Marina Freudenberg (Max Planck Institute for Immunobiology, Freiburg). IFN-γ^-/- ^mice [8], IFN-receptor deficient mice (IFNAR, [10]), (IFNGR, [9]) and STAT-1^-/- ^mice [41] were provided by Dr Thomas Kolbe (University of Veterinary Medicine, Vienna). RAG-1^-/- ^mice [13] were provided by Dr Heike Weighardt (Technische Universitaet Muenchen, Munich). JHT mice [14] were provided by Dr Ari Waisman (Johannes Gutenberg-Universität Mainz, Mainz, Germany). Germ-free C57BL/6 mice were provided from three independent facilities, namely, University of Zürich, Zürich, Switzerland (Mr Rudolf Jörg), the Gulbenkian Institute of Science, Oeiras, Portugal (Dr Jocelyne Demengeot) and the Karolinska Institute, Stockholm. CD1d^-/- ^mice [21] were provided by Professor Luc van Kaer (Vanderbilt University Medical Center, TN, USA). Jα18^-/- ^mice [22] were provided by Dr Maria C. Leite-de-Moraes, Hôpital Necker, Paris, France. MAVS/CARDIF-deficient mice [30] were provided by Dr Jürg Tschopp (Lausanne, Switzerland). MHC Aβ^-/- ^mice [23] were provided by Dr Jocelyne Demongeot (Gulbenkian Institute of Sciences, Oeiras, Portugal). TAP1^-/- ^mice [24] were purchased from the Jackson Laboratory (Maine, US). For all assays, wild-type C57BL/6 mice from the same donor facility were used as controls. To control germ-free C57BL/6 mice, C57BL/6 mice from colonies maintained at SPF level from the same suppliers were used. All the above mice were on the C57BL/6J background. SWR/J, SJL, C57BR/J, C57L/J, RIIIS/J, A/J and C57BR/cdJ strains were purchased from The Jackson Laboratory (Maine, USA). In most cases, liver and kidneys were removed at the donor animal facility, immediately fixed in cold TBS/4% paraformaldehyde and shipped at 4°C by the quickest route to the Institute for Genetics in Cologne, Germany.

### Tissue preparation

For paraffin sections, mouse tissues were fixed in TBS/4% paraformaldehyde at 4°C and dehydrated through an ethanol series at 4°C (50%, 70%, 90% and 96%). Tissues were then transferred into isopropanol and finally into a paraffin:isopropanol (1:1) solution at 60°C. The isopropanol was evaporated and fresh paraffin was then replenished several times at 60°C before the tissues (in paraffin) were moved to room temperature. The embedded tissues were cut with a microtome RM 2065 (Leica Microsystems, Wetzlar, Germany) into 6 μm thick serial sections. For cryosections, mouse liver was snap-frozen in liquid nitrogen and cut into consecutive serial sections (6 μm) using a cryotome CM 3050 S (Leica Microsystems).

### Immunohistochemistry

Paraffin sections were de-waxed in xylene and post-fixed in 4% paraformaldehyde (1 h, RT). For Irga6 or Irgm3 staining, protein epitopes were demasked (10 min, boiling) in 10 mM citrate buffer (pH 6.0). For F4/80 or CD3 staining, protein epitopes were demasked by 0.1% Trypsin (SIGMA Type II) solution (0.1% CaCl_2_, pH7.8) for 30mins at 37°C or in 1 mM EDTA (pH8.0) for 10 min at 100°C, respectively. Sections were then saturated with quenching buffer (0.3% H_2_O_2, _20 min). After phosphate buffered saline (PBS) washing, the sections were probed either with rabbit anti-Irga6 antiserum 165/3 [42] or mouse anti-Irgm3 mAb (BD Transduction Laboratory) in DAKO diluent. HRP-staining was carried out with the horseradish peroxidase (HRP)-substrate kit HistoGreen (Linaris, Wetzlar, Germany). Nuclei were counterstained with Nuclear Fast Red. For Irga6 and F4/80 or CD3 double staining, Irga6 was first probed with 165/3 and stained with HRP-substrate AEC (Sigma, MO, USA). The sections were then washed with PBS (1 h) and subsequently probed with anti-F4/80 mAb (Serotec, NC, USA) or anti-CD3 mAb (Vector Laboratories, Linaris). The AP-staining was then carried out with AP-substrate kit III (Vector laboratory, Linaris). For staining of cryo-sections, sections were fixed first with cold acetone (10 min), before probing with anti-Irga6 antiserum 165/3 or anti-TCR Vβ8.1/8.2 mAb (BD Pharmingen, CA, USA). The sections were then stained with the HRP-substrate kit HistoGreen for Irga6 or with AP-substrate kit III for TCR Vβ 8. Samples were analysed using Zeiss Axioplan II microscope (Zeiss, Jena, Germany) equipped with SPOT RT slider digital camera (Diagnostic Instruments, MI, USA).

### Quantitation of Irga6 expression foci in histological sections

Irga6 high expression foci were counted on stained 6 μm histological sections using 100× total magnification. Sections 300 μm apart were examined in order to avoid repeated counts of the same high expression patch. A total of 30 fields were counted from each organ, from which the mean number of high expression patches per field was estimated. The raw data from all such determinations are presented in Additional file 1: Table S1.

### Laser microdissection

Consecutive serial cryosections (6 μm) for liver were prepared using a cryotome CM 3050 S (Leica Microsystems) and sections were carried on PALM MembraneSlides (P.A.L.M. Microlaser Technologies, Bernried, Germany), which was covered with a polyethylene naphthalate membrane. In order to avoid the RNA degradation caused by the staining of sections with antibody, the following procedure was followed. Consecutive sections were numbered (1, 2, 3, 4...). All sections with odd-numbers (1, 3...) were on one slide (named 'A') and the sections with even numbers (2, 4...) on another slide (named 'B'). Both slides were then fixed in 70% ethanol (-20°C). The B slide was stored at -80°C and the A slide was stained for Irga6 with 165/3 using histogreen substrate. Geographical locations of Irga6 focal expression on the A slide were recorded photographically. The B slide was then stained only with Nuclear Fast Red, dehydrated in an ethanol series (70%, 96%, 100% 2 min each) and dried at 50°C. Irga6 focal expression was located on the B slide by referring to the photos of adjacent sections on the A slide. The Irga6 expression foci and non-focus region on the B slides were then collected by laser microdissection (LMD) equipment generously made available by Dr Margarete Odenthal (Institute for Pathology, University of Cologne, Cologne, Germany) consisting of an Interface Microbeam Mini laser (P.A.L.M Microlaser Technologies) and an Axiovert 135 microscope (Zeiss). Total RNA from the dissected samples (50-100 foci) was extracted with the RNeasy Micro kit (QIAGEN, Hilden, Germany) and cDNA was synthesized with half of the total RNA with the Superscript First-Strand Synthesis System for RT-PCR kit (Invitrogen, CA, USA).

### Real-time PCR

PCR was carried out in a Light Cycler I System (Roche, Berlin, Germany) using a LightCycler SYBR Green I PCR kit (Roche). The 5' primers specific for Irga6 1A and 1B together with the common 3' primer on the coding exon were used to detect expression level of Irga6-1A and Irga6-1B transcript forms as target genes [4]. Mouse GAPDH gene was used as a reference gene and input control (for primers see Additional file 4: Table S2). 2-4 μL of cDNA from materials collected by LMD was used as templates. Primer efficiency for Irga6-1A, -1B or mGAPDH was determined using liver cDNA dilution series (1, 1/10, 1/100...) as templates as described previously [43]. The proportional increase in Irga6-1A or -1B expression for 'foci' to 'non-foci' was then determined according to [43]. Melting curve analyses were performed in order to verify the amplification specificity. Each sample was tested in duplicate or triplicate. In order to determine whether the final PCR products amplified for Irga6-1A were -1A specific, 1A PCR products were cloned in to pGEM-T-easy (Promega, CA, USA) vector and sequenced. The percentage of -1A specific clones was then calculated. For Irga6 high expression foci, all clones amplified for Irga6-1A were -1A, while for the non-focal material, only 5.9% of clones amplified for Irga6-1A were -1A. The directly determined enrichment factor of 27.9 for Irga6-1A in Irga6 high expression foci (Figure 2C) can, therefore, be multiplied a factor of 16.9 (100/5.9) in order to give a relative enrichment of over 470 in Irga6-1A compared with non-focal tissue.

### Nested-RT-PCR

Total RNA from mouse liver or lymph nodes was extracted using the RNeasy Mini kit (QIAGEN) and cDNA was synthesized using the Superscript First-Strand Synthesis System for RT-PCR kit (Invitrogen). One mcrolitre cDNA from the liver and T cells or 2-4 μL of cDNA from laser dissected materials were used in each of the following RT-PCR reactions as templates. Primers located in different exons were used to generate specific products only from cDNA but not from genomic DNA (Additional file 4: Table S2). For nested-PCR, 1 μL PCR product of the first round of PCR was used as template for the second PCR, making use of a nested primer and the common 3' or 5' primer. The cycle number was always 45 for each round of PCR. All final PCR-products were verified by sequencing.

### TCR repertoire screening

In order to determine the TCR Vβ usage and Vα14 junctional diversity, nested-RT-PCR was performed. cDNA from more than 50 Irga6 cored patches collected by LMD or cDNA of 1000 lymphocytes from mesenteric and cervical lymph nodes were synthesized and nested-RT-PCR was performed as described in [4] The Vβ8 and Cβ primers [44] Vα14 and Cα primers [16], Vα2, Vα8, Vα17 primers [45] were described before (Additional file 4: Table S2). The final products of nested-PCR were cloned into pGEM-T-easy (Promega) and sequenced. The sequences were compared to classical TCR V sequences in the database online using IMGT/V-QUEST http://imgt.cines.fr/. Clones with a sequence identity of more than 95% to known TCR V sequences were identified as correct TCR V clones. Clones were classified into groups with same junctions. Representatives from each group were selected aligned and analysed with free software GeneDoc (Version 2.6.002; http://www.nrbsc.org) and Vector NTI (Version 9, Invitrogen). See Additional file 5: Table S3 for complete junction sequence data.

## Abbreviations

α-GalCer: alpha-galactosyl ceramide; F: foci; NF: non-focus region; iNKT: invariant natural killer-like T cell; FN: interferon; IFNAR: IFN type I receptor; IFNGR: IFN type II receptor; IL: interleukin; HRP: horseradish peroxidase; IRG: immunity-related GTPases; MHC: major histocompatibility; MMTV: mouse mammary tumour virus; PBS: phosphate buffered saline; PCR: polymerase chain reaction; qRT-PCR: quantitative reverse transcription PCR; TCR: T-cell receptor; TLR: Toll-like receptor.

## Competing interests

The authors declare that they have no competing interests.

## Authors' contributions

JZ and JCH identified the problem and conceived the experimental approach. JZ performed all the experimental work, counted the histological data and prepared the figures. JCH drafted the manuscript, which was completed collaboratively by JZ and JCH. Both authors read and approved the final manuscript.

## Supplementary Material

Additional file 1 **Table S1 - Quantification of Irga6 focal expression patches in liver and kidneys of various mouse strains**. Focal Irga6 expression was quantified on stained histological sections of liver and kidney from strains of mice listed, as described in Materials and Methods. Each value in the table is the mean number of expression foci per microscope field averaged over 30 fields. WT = wild type; KO = knock-out, IFN R are mice lacking IFN-type I (IFNAR) and type II (IFNGR) receptors respectively; SPF = specific pathogen free; GF = germ-free; Portugal, Sweden and Switzerland refer to the national origins of three independent germ-free C57BL/6 strains. Click here for file

Additional file 2 **Figure S1 - Irga6 is over-expressed in the kidney of RAG and JHT mice**. Paraffin sections from kidneys of RAG or JHT mice were probed for Irga6 protein (Green). Nuclei were counterstained in red. Kidneys from WT control mice were also included. Click here for file

Additional file 3 **Figure S2 - Irgm3 is co-expressed at Irga6 expression foci in liver and kidney**. Serial paraffin sections (6 μm) of organs from C57BL/6 adult mice were prepared. In each case, two adjacent serial sections were probed for Irga6 (1, green) and Irgm3 protein (2, green) respectively. Frames show enlarged images. Nuclei were counter-stained in red. Click here for file

Additional file 4 **Table S2 - List of primers used in RT-PCR and Real time PCR**. Click here for file

Additional file 5 **Table S3 - Sequence comparison of identified TCR clones**. Sequences of identified TCR Vβ8 (A) and Vα14/11 (B) clones from Irga6 liver cored patches, and sequences of identified TCR Vα11 (C) clones from lymph nodes were compared and classified into different junction types. The number of clones belonging to each junction type was listed. Vβ, Dβ, Jβ, Vα and Jα subfamily names were labeled (' - ' means not identifiable). TCR Vβ-N(D)N-Jβ or TCR Vα-N-J junctional sequences were displayed. 'Short' indicates that the length of the sequence was too short to identify the entire junctional region. Click here for file
