# Supplementary material for: Spontaneous focal activation of invariant natural killer T (iNKT) cells in mouse liver and kidney
Source: BMC Biol. 2010 Nov 30;8:142. doi: 10.1186/1741-7007-8-142 (PMC3016249; doi:10.1186/1741-7007-8-142)
Supplement: Additional file 1 — Table S1 - Quantification of Irga6 focal expression patches in liver and kidneys of various mouse strains. Focal Irga6 expression was quantified on stained histological sections of liver and kidney from strains of mice listed, as described in Materials and Methods. Each value in the table is the mean number of expression foci per microscope field averaged over 30 fields. WT = wild type; KO = knock-out, IFN R are mice lacking IFN-type I (IFNAR) and type II (IFNGR) receptors respectively; SPF = specific pathogen free; GF = germ-free; Portugal, Sweden and Switzerland refer to the national origins of three independent germ-free C57BL/6 strains. [file 1741-7007-8-142-S1.doc]

| Mice strains | | liver | | kidney |
| --- | --- | --- | --- | --- |
|  |  | patch | cored patch |  |
| IFN-γ | WT | 10.2 | 1.7 | 4.2 |
|  | KO | 8.8 | 0 | 0 |
| IFN R | WT | 7.7 | 0.7 | 3.8 |
|  | IFNAR | 5.1 | 0.6 | 2.2 |
|  | IFNGR | 5 | 0 | 0.3 |
| STAT1 | WT | 8.8 | 1.5 | 8.4 |
|  | KO | 0 | 0 | 0 |
| RAG1 | WT | 12.7 | 1.4 | 6 |
|  | KO | 4.9 | 0 | ND |
| JHT | WT | 9.4 | 1.4 | 12.4 |
|  | KO | 10.2 | 4.1 | ND |
| TLR2/4 | WT | 11 | 0.8 | 6.9 |
|  | KO | 11.7 | 1.3 | 7.4 |
| TLR9 | WT | 9.1 | 1.1 | 9.8 |
|  | KO | 9.2 | 1.1 | 11.3 |
| MyD88 | WT | 8.8 | 2 | ND |
|  | KO | 13.1 | 1.3 | ND |
| Portugal | SPF | 8.5 | 1.3 | 3.5 |
|  | GF | 9.9 | 1.3 | 17.6 |
| Switzerland | SPF | 8.3 | 2.3 | 6.7 |
|  | GF | 7.7 | 2.1 | 8.1 |
| Sweden | SPF | 9.2 | 1 | 6.5 |
|  | GF | 8.3 | 0.9 | 5 |
| CD1d | WT | 11.6 | 1.6 | 5.1 |
|  | KO | 11.6 | 0.03 | 0.17 |
| Jα 18 | WT | 11 | 1.3 | 5 |
|  | KO | 11.7 | 0.16 | 0.41 |
| MHC II Aβ | WT | 8.94 | 1.06 | 3.6 |
|  | KO | 10.55 | 1.4 | 7.2 |
| MAVs | WT | 9 | 1 | 4.6 |
|  | KO | 7.2 | 0.8 | 2.9 |

| Mice strains  (Jackson laboratory) | liver | | kidney |
| --- | --- | --- | --- |
|  | patch | cored patch |  |
| C57BL/6J | 11.7 | 1 | 3.7 |
| A/J | 7.9 | 0.7 | 2.3 |
| C57BR/cdj | 11.5 | 1.23 | 1.7 |
| SWR/J | 0.77 | 0.2 | 0.17 |
| SJL | 4.5 | 0.27 | 0.8 |
| RIIIS/J | 5.4 | 0 | 0.2 |
| C57BR/L | 9.7 | 0.16 | 0.37 |
| C57L/J | 7.8 | 0.2 | 0.03 |
| TAP1 KO | 8.68 | 1.26 | 4 |
