# Supplementary figures and images for: Spontaneous focal activation of invariant natural killer T (iNKT) cells in mouse liver and kidney
Source: BMC Biol. 2010 Nov 30;8:142. doi: 10.1186/1741-7007-8-142 (PMC3016249; doi:10.1186/1741-7007-8-142)

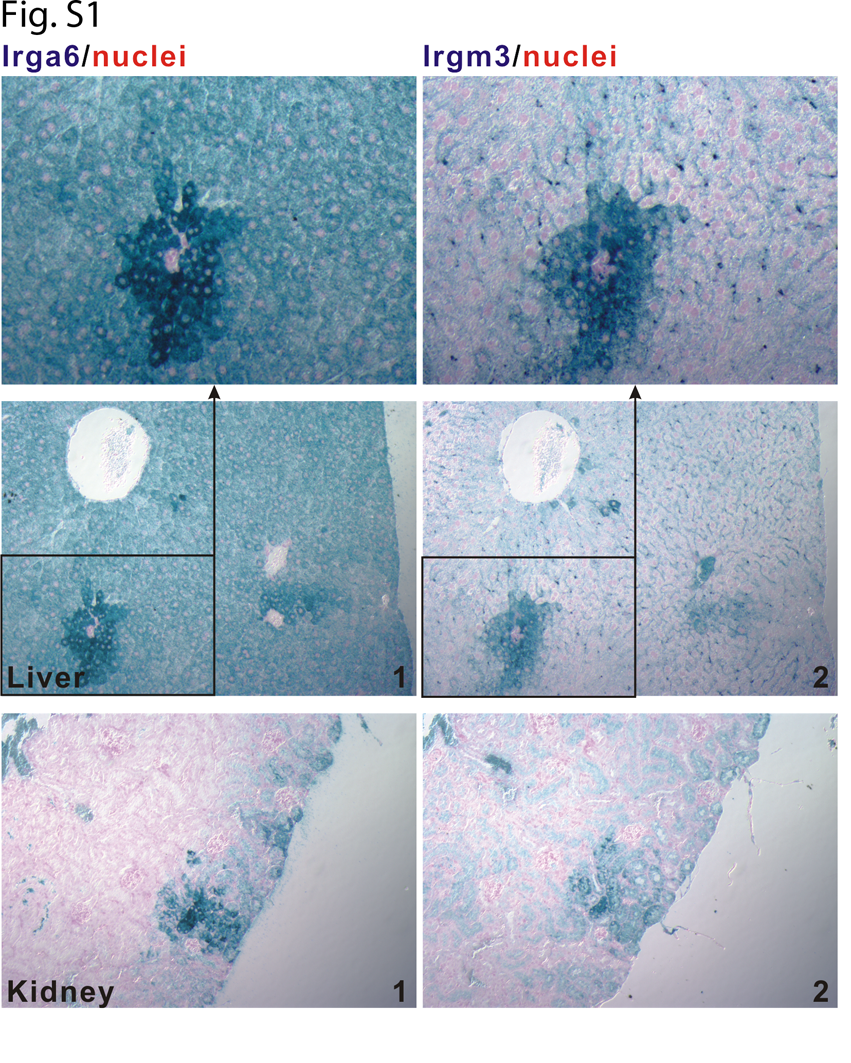

Supplement: Additional file 3 — Figure S2 - Irgm3 is co-expressed at Irga6 expression foci in liver and kidney. Serial paraffin sections (6 μm) of organs from C57BL/6 adult mice were prepared. In each case, two adjacent serial sections were probed for Irga6 (1, green) and Irgm3 protein (2, green) respectively. Frames show enlarged images. Nuclei were counter-stained in red. [file 1741-7007-8-142-S3.tiff]
