# Supplementary material for: Spontaneous focal activation of invariant natural killer T (iNKT) cells in mouse liver and kidney
Source: BMC Biol. 2010 Nov 30;8:142. doi: 10.1186/1741-7007-8-142 (PMC3016249; doi:10.1186/1741-7007-8-142)
Supplement: Additional file 4 — Table S2 - List of primers used in RT-PCR and Real time PCR. [file 1741-7007-8-142-S4.doc]

| **primer name** | **primer sequence 5’ to 3’** | **product size** |
| --- | --- | --- |
| **Irga6 1A** | **TGCTTCCTGAAGCTGAACTA** | **969bp** |
|  | **CAGAGAAGGGATGATATTCAC** |  |
| **Irga6 1B** | **ACCGAGGGCTATTCCTCTCA** | **972bp** |
|  | **CAGAGAAGGGATGATATTCAC** |  |
| **IFNγ** | **CTGAGACAATGAACGCTACACA** | **507bp** |
|  | **TTATTGGGACAATCTCTTCCC** |  |
| **IFNγ nest** | **ACTGGCAAAAGGATGGTGAC** | **332bp** |
|  | **TTATTGGGACAATCTCTTCCC** |  |
| **TCR β chain** | **GGT(G/T)T(A/C/T)(C/T)TGGTA(C/T)(A/C/T)(A/G)(A/C/G/T) CA** | **450bp** |
|  | **TCAGGCAGTAGCTATAA** |  |
| **TCR β chain nest** | **GGT(G/T)T(A/C/T)(C/T)TGGTA(C/T)(A/C/T)(A/G)(A/C/G/T) CA** | **280bp** |
|  | **GGTGGAGTCACATTTCT** |  |
| **TCR Vα14 chain** | **CTAAGCACAGCACGCTGCACA** | **350bp** |
|  | **GAAGCTTGTCTGGTTGCTCCAG** |  |
| **TCR Vα14 chain nest** | **CTAAGCACAGCACGCTGCACA** | **220bp** |
|  | **TCGGTGAACAGGCAGAGGGTG** |  |
| **TCR Vα2 chain** | **TGCAGTTATGAGGACAGCACTT** | **480bp** |
|  | **GAAGCTTGTCTGGTTGCTCCAG** |  |
| **TCR Vα2 chain nest** | **TGCAGTTATGAGGACAGCACTT** | **350bp** |
|  | **TCGGTGAACAGGCAGAGGGTG** |  |
| **TCR Vα8 chain** | **ACGCCACTCTCCATAAGAGCA** | **360bp** |
|  | **GAAGCTTGTCTGGTTGCTCCAG** |  |
| **TCR Vα8 chain nest** | **ACGCCACTCTCCATAAGAGCA** | **230bp** |
|  | **TCGGTGAACAGGCAGAGGGTG** |  |
| **TCR Vα17 chain** | **TTCCATCGGACTCATCATCAC** | **340bp** |
|  | **GAAGCTTGTCTGGTTGCTCCAG** |  |
| **TCR Vα17 chain nest** | **TTCCATCGGACTCATCATCAC** | **210bp** |
|  | **TCGGTGAACAGGCAGAGGGTG** |  |
| **mouse GAPDH** | **GTCTACATGTTCCAGTATGACTCCACTCACGG** | **837 bp** |
|  | **GTTGCTGTAGCCGTATTCATTGTCATACCAGG** |  |
