# Supplementary material for: Spontaneous focal activation of invariant natural killer T (iNKT) cells in mouse liver and kidney
Source: BMC Biol. 2010 Nov 30;8:142. doi: 10.1186/1741-7007-8-142 (PMC3016249; doi:10.1186/1741-7007-8-142)
Supplement: Additional file 5 — Table S3 - Sequence comparison of identified TCR clones. Sequences of identified TCR Vβ8 (A) and Vα14/11 (B) clones from Irga6 liver cored patches, and sequences of identified TCR Vα11 (C) clones from lymph nodes were compared and classified into different junction types. The number of clones belonging to each junction type was listed. Vβ, Dβ, Jβ, Vα and Jα subfamily names were labeled (' - ' means not identifiable). TCR Vβ-N(D)N-Jβ or TCR Vα-N-J junctional sequences were displayed. 'Short' indicates that the length of the sequence was too short to identify the entire junctional region. [file 1741-7007-8-142-S5.doc]

| Junction Type | Number of clones | Dβ | Jβ | V 8.1 region | NDN region | J region |
| --- | --- | --- | --- | --- | --- | --- |
| 1 | 4 | - | 1.4 | ATTTCTGTGCCAGCAGTGAT | AGGCAG | AACGAAAGATTATTTTTCGG |
| 2 | 1 | - | 1.4 | ATTTCTGTGCCAGCAGTGAT | GTCCAG | AACGAAAGATTATTTTTCGG |
|  |  |  |  |  |  |  |
| Junction Type | Number of clones | Dβ | Jβ | V8.2 region | NDN region | J region |
| 1 | 2 | 1.1 | 1.1 | GTGTACTTCTGTGCCAGCGGTG | GGGGGCTTG | CAAACACAGAAGTCTTCTTT |
| 2 | 14 | 1.1 | 2.7 | GTGTACTTCTGTGCCAGCGG | GGACAGC | GAACAGTACTTCGGTCCCGG |
| 3 | 3 | 1.1 | 2.7 | GTGTACTTCTGTGCCAGCGGTGATG | CGGACAA | CTCCTATGAACAGTACTTCGGTCCCGG |
| 4 | 2 | 2.1 | 2.3 | GTGTACTTCTGTGCCAGCGG | GGGGACATCT | AGTGCAGAAACGCTGTATTT |
| short | 3 |  |  |  |  |  |
|  |  |  |  |  |  |  |
| Junction Type | Number of clones | Dβ | Jβ | V8.3 region | NDN region | J region |
| 1 | 1 | 1.1 | 1.1 | TTGTACTTCTGTGCCAGCAG | GGACAG | CACAGAAGTCTTCTTTGGTA |
| 2 | 3 | 1.1 | 2.1 | TTGTACTTCTGTGCCAGCAG | AAGGACAGGGGG | CTATGCTGAGCAGTTCTTCG |
| short | 1 |  |  |  |  |  |

| Junction type | Number of clones | Vα | Jα | V region | N region | J region |
| --- | --- | --- | --- | --- | --- | --- |
| 1 | 14 | 14 | 18 | ACCTACATCTGTGTGGTGGG |  | AGATAGAGGTTCAGCCTTAG |
| 2 | 3 |  |  | ACCTACATCTGTGTGGTG | TC | AGATAGAGGTTCAGCCTTAG |
| 3 | 18 |  |  | ACCTACATCTGTGTGGTGGG | G | GATAGAGGTTCAGCCTTAG |
| 4 | 8 |  |  | ACCTACATCTGTGTGGTGGG | T | GATAGAGGTTCAGCCTTAG |
| 5 | 24 |  |  | ACCTACATCTGTGTGGTGGG | C | GATAGAGGTTCAGCCTTAG |
| 6 | 3 |  |  | ACCTACATCTGTGTGGTGG | TC | GATAGAGGTTCAGCCTTAG |
|  | 2 | 11 | 49 | TTACTTCTGTGCTGCTGAGG | TCTT | CACGGGTTACCAGAACTTCT |

| Number of clones | Jα | Vα11 region | N region | J region |
| --- | --- | --- | --- | --- |
| 1 | 12 | ACTTACTTCTGTGCTGCTGA | AACC | GGGACTGGAGGCTATAAAGT |
| 5 | 40 | ACTTACTTCTGTGCTGCTG | GTG | ATACAGGAAACTACAAATAC |
| 4 | 22 | ACTTACTTCTGTGCTG | TGCTGCCG | CATCTTCTGGCAGCTGGCAA |
| 5 | 48 | ACTTACTTCTGTGCTGCTG | CCAATT | CCAACTATGGAAATGAGAAA |
| 2 | 31 | ACTTACTTCTGTGCTGCTG | GGCCTTT | AATCTTCTTTGGTGATGGGA |
| 2 | 45 | ACTTACTTCTGTGCTGC | CTC | GAATACAGAAGGTGCAGATA |
| 2 | 13 | ACTTACTTCTGTGCTGCTGAGG | CGGGGG | ATTCTGGGACTTACCAGAGG |
| 3 | 23 | ACTTACTTCTGTGCTGCTGAGG | CC | TATAACCAGGGGAAGCTTAT |
| 5 | 33 | ACTTACTTCTGTGCTGTTGAG | CTC | GATAGCAACTATCAGTTGAT |
| 1 | 21 | ACTTACTTCTGTGCTGCTGAG | AG | GTCTAATTACAACGTGCTTT |
| 5 | 21 | ACTTACTTCTGTGCTGTTGAG | GCGTA | TGTCTAATTACAACGTGCTTT |
| 5 | 21 | ACTTACTTCTGTGCTGCTGAGG | CGG | CTAATTACAACGTGCTTT |
| 1 | short |  |  |  |
